# Supplementary material for: Zebrafish Oxr1a Knockout Reveals Its Role in Regulating Antioxidant Defenses and Aging
Source: Genes (Basel). 2020 Sep 24;11(10):1118. doi: 10.3390/genes11101118 (PMC7598701; doi:10.3390/genes11101118)
Supplement: Supplementary file 1 [file genes-11-01118-s001.zip › genes-921171-Supplementary files/Table S1.docx]

**Table S1. Sequences of primers used in this study**

| Gene | Primer sequence (5'-3') | Usage |
| --- | --- | --- |
| *oxr1a*-Cas-F | TAATACGACTCACTATAGGTTCTGGAAAAAGGCTGTGGTTTTAGAGCTAGAAATAGC | Gene knockout |
| *oxr1a*-Cas-R | aaaaaaagcaccgactcggt |  |
| *oxr1a*-F1 | AAAAAAAGCATAAAAGTACGCA | Mutation analyses |
| *oxr1a*-R1 | ATTGACGACACCAACAGGATA |  |
| *oxr1a-*F2 | GAAAAAGGCTGTGAGGAGTAC | Mutation analyses |
| *oxr1a-*R2 | TGAGAACACGGCTTCAAGAT |  |
| *oxr1a*-PAGE-F | TTGCTGGTGACACCCAACAA | Mutation analyses |
| *oxr1a*-PAGE-R | TCTTTGTAGACAGCGGCAGA |  |
| *oxr1a*-probe-F | AGCAGCAACCTGAGGAAAGCT | WISH |
| *oxr1a*-probe-R | ATTGACGCTGCACACGGCTT |  |
| *gstp1*-probe-F | TGAAACGCACTTCACTCAGC | WISH |
| *gstp1*-probe-R | CAGTAAGTGTCGTTGTTGGAG |  |
| *gstp2*-probe-F | ACATTTCAGGAGCAGCAACT | WISH |
| *gstp2*-probe-R | CACTGTTTGCCGTTGCCGTT |  |
| *prdx1*-probe-F | TATCGCGAGACTTGAGCACG | WISH |
| *prdx1*-probe-R | GCTGCAGAGTCTAGTGCCTT |  |
| Q-*sod1*-F | ACCGGCACCGTCTATTTCAA | Real-time PCR |
| Q-*sod1*-R | AGCATGGACGTGGAAACCAT |  |
| Q-*sod2*-F | TAGGTCTGTTGGTTGGTCGC | Real-time PCR |
| Q-*sod2*-R | ATGTTGCATGGTGCTTGCTG |  |
| Q-*sod3a*-F | TGAAGTCTCTCCCATCCCAA | Real-time PCR |
| Q-*sod3a*-R | CCCTGACTGAGGTCTCCGTA |  |
| Q-*sod3b*-F | TGGCCCAAAGGAAAAGCTGA | Real-time PCR |
| Q-*sod3b*-R | GGATTCCAGTGACTGCCGAA |  |
| Q-*gpx1a*-F | TTTACGACCTGTCCGCGAAA | Real-time PCR |
| Q-*gpx1a*-R | CTGTTGTGCCTCAAAGCGAC |  |
| Q-*gpx1b*-F | TTCCCAAGCGATGAGCCAAT | Real-time PCR |
| Q-*gpx1b*-R | TTGATGTCTCCGTCGATGCC |  |
| Q-*gpx2*-F | GGCTTGTAGTCCTGGGCTTT | Real-time PCR |
| Q-*gpx2*-R | TCACTCCCGTTTACAACGCA |  |
| Q-*gpx3*-F | gcagttcatcccgttctctca | Real-time PCR |
| Q-*gpx3*-R | ttctccaggttcttgtttccc |  |
| Q-*gpx4a*-F | TGCGTTTCTTAGGGTCTGCT | Real-time PCR |
| Q-*gpx4a*-R | TCTCAGAGTACTTGGCGTGC |  |
| Q-*gpx4b*-F | GAAAGCAGGAGCCTGGAAGT | Real-time PCR |
| Q-*gpx4b*-R | TTGGATCGTCCATTGGTCCG |  |
| Q-*gpx7*-F | CGCAGAGTCTACGGGGTTTC | Real-time PCR |
| Q-*gpx7*-R | CAGGGTTCGCAGATGAGGAT |  |
| Q-*gpx8*-F | ACACGCCCACAAAAACTTCA | Real-time PCR |
| Q-*gpx8*-R | TTTCCCTCGGTATTTCTCCA |  |
| Q-*cat*-F | AAAATGGGGGCCTTTGCATAC | Real-time PCR |
| Q-*cat*-R | GCAGAAAGGACGGCAAACATT |  |
| Q-*cdkn1a*-F | GTCGTCAGAGGCAGCAGAAA | Real-time PCR |
| Q-*cdkn1a*-R | GAGTGAACGTAGGATCCGCT |  |
| Q-*casp8l2*-F | AGATTTGCCGTCAGCACAGA | Real-time PCR |
| Q-*casp8l2*-R | TGAATCCGAATGCTGGCTGT |  |
| Q-*fas*-F | CAAACACGGTCTGTGGGTGT | Real-time PCR |
| Q-*fas*-R | CTTTGGCATCATGGCACACT |  |
| Q-*casp3b*-F | ACAACACCAGAAGCAGGACTT | Real-time PCR |
| Q-*casp3b*-R | TTTGCATCGCTTTGTCTGGC |  |
| Q*-pmaip1*-F | GGCTGTTTTGAGCCGTGTTT | Real-time PCR |
| Q*-pmaip1*-R | TGTCGGTTTCCAGTCCTTGG |  |
| Q-*gadd45aa*-F | GTCCCTCAATGTGGACCCTG | Real-time PCR |
| Q-*gadd45aa*-R | CATGTGGAACCGTGACCAGA |  |
| Q-*gadd45ba*-F | CGCTGAACTATTCGCGATGC | Real-time PCR |
| Q-*gadd45ba*-R | TCCGCTACCAAAAACGACCA |  |
| Q-*gadd45bb*-F | CAAAGGAGCATCTGGGTGGT | Real-time PCR |
| Q-*gadd45bb*-R | GAGCTTCTCCGCTGTAGACC |  |
| Q-*serpine1*-F | TACTCCCGTATGGCTGTGGA | Real-time PCR |
| Q-*serpine1*-R | CAGACACGTCTCCTCGGTTC |  |
| Q-*serpine3*-F | TACGGCAAATCAGCACAGGT | Real-time PCR |
| Q-*serpine3*-R | CCTCTGACCCTCCACCCATA |  |
| Q- *LOC100535166*-F | CCGAAATCCCAGATGACGAC | Real-time PCR |
| Q- *LOC100535166*-R | CAGGCTCGCAGTAAACACGA |  |
| Q-*sesn2*-F | AGGCAGTTTCGACACTCTGA | Real-time PCR |
| Q-*sesn2*-R | GTGCGATGTGTGTGTGAAAAAC |  |
| Q-*nfe2l2a*-F | GGCGATCCTCCTGTAAACCC | Real-time PCR |
| Q-*nfe2l2a*-R | CGAAGGATCCGTCTTCGGTT |  |
| Q-*nfe2l2b*-F | ATCCCTTGTTGTCTGCCCTG | Real-time PCR |
| Q-*nfe2l2b*-R | TGGATCGGGGAGGTTAGGTT |  |
| Q-*keap1a*-F | GAGGGGTCACGGTTACTTGG | Real-time PCR |
| Q-*keap1a*-R | GGACAAACGTCTCGAAGGGT |  |
| Q-*keap1b*-F | TGGAGGCTTATAACGCGGATT | Real-time PCR |
| Q-*keap1b*-R | AGGAACACACGCAGAGGAAC |  |
| Q*-oxr1a*-F | CACCTGCTGCCAAGACAAA | Real-time PCR |
| Q*-oxr1a*-R | GAACATACAAGACCTGACCCG |  |
| Q-*eef1a1l1*-F | gcttctctacctaccctcctct | Real-time PCR |
| Q-*eef1a1l1*-R | caccaccgattttcttctca |  |
