# Supplementary material for: Zebrafish Oxr1a Knockout Reveals Its Role in Regulating Antioxidant Defenses and Aging
Source: Genes (Basel). 2020 Sep 24;11(10):1118. doi: 10.3390/genes11101118 (PMC7598701; doi:10.3390/genes11101118)
Supplement: Supplementary file 1 [file genes-11-01118-s001.zip › genes-921171-Supplementary files/Table S4.docx]

**Table S4. Top pathways affected by H_2_O_2_ stress in zebrafish**

| **Pathway Identification (ID)** | | **DEGs with PATHWAY Annotation (328)** | **All Genes with Pathway Annotation (13482)** | ***P* value** | ***Q* value** |
| --- | --- | --- | --- | --- | --- |
| **WT zebrafish during H_2_O_2_ stress** | | | | | |
| ko04216 | Ferroptosis | 12 | 72 | 1.59 × 10^−10^ | 3.76 × 10^−8^ |
| ko04978 | Mineral absorption | 11 | 76 | 4.50 × 10^−9^ | 5.31 × 10^−7^ |
| ko04217 | Necroptosis | 16 | 311 | 3.92 × 10^−6^ | 3.08 × 10^−4^ |
| ko04668 | TNF signaling pathway | 13 | 235 | 1.52 × 10^−5^ | 8.97 × 10^−4^ |
| ko00020 | Citrate cycle (TCA cycle) | 7 | 73 | 5.04 × 10^−5^ | 1.98 × 10^−3^ |
| ko04657 | IL−17 signaling pathway | 11 | 189 | 4.40 × 10^−5^ | 1.98 × 10^−3^ |
| ko05204 | Chemical carcinogenesis | 8 | 110 | 1.07 × 10^−4^ | 3.60 × 10^−3^ |
| ko04915 | Estrogen signaling pathway | 11 | 231 | 2.62 × 10^−4^ | 7.72 × 10^−3^ |
| ko00620 | Pyruvate metabolism | 7 | 98 | 3.24 × 10^−4^ | 8.49 × 10^−3^ |
| Ko00670 | One carbon pool by folate | 9 | 168 | 4.06 × 10^−4^ | 9.59 × 10^−3^ |
| ko05164 | Influenza A | 14 | 375 | 4.83 × 10^−4^ | 1.04 × 10^−2^ |
| ko04380 | Osteoclast differentiation | 10 | 215 | 5.98 × 10^−4^ | 1.16 × 10^−2^ |
| ko05211 | Renal cell carcinoma | 8 | 143 | 6.40 × 10^−4^ | 1.16 × 10^−2^ |
| ko05145 | Toxoplasmosis | 10 | 237 | 1.30 × 10^−3^ | 2.12 × 10^−2^ |
| ko00980 | Metabolism of xenobiotics by cytochrome P450 | 6 | 92 | 1.40 × 10^−3^ | 2.20 × 10^−2^ |
| ko05222 | Small cell lung cancer | 8 | 175 | 2.34 × 10^−3^ | 3.45 × 10^−2^ |
| ko03460 | Fanconi anemia pathway | 9 | 221 | 2.78 × 10^−3^ | 3.86 × 10^−2^ |
| ko03420 | Nucleotide excision repair | 9 | 228 | 3.42 × 10^−3^ | 4.49 × 10^−2^ |
| ko00982 | Drug metabolism −cytochrome P450 | 5 | 79 | 3.98 × 10^−3^ | 4.49 × 10^−2^ |
|  | | | | | |
| ***oxr1a^−/−^* mutant zebrafish during H_2_O_2_ stress** | | | | | |
| ko04216 | Ferroptosis | 13 | 72 | 1.61 × 10^−8^ | 4.39 × 10^−6^ |
| ko04657 | IL−17 signaling pathway | 16 | 189 | 1.53 × 10^−5^ | 2.09 × 10^−3^ |
| ko00920 | Sulfur metabolism | 5 | 19 | 7.26 × 10^−5^ | 2.63 × 10^−3^ |
| ko00980 | Metabolism of xenobiotics by cytochrome P450 | 10 | 92 | 7.80 × 10^−5^ | 2.63 × 10^−3^ |
| ko04621 | NOD−like receptor signaling pathway | 35 | 691 | 3.18 × 10^−5^ | 2.63 × 10^−3^ |
| ko04668 | TNF signaling pathway | 17 | 235 | 6.31 × 10^−5^ | 2.63 × 10^−3^ |
| ko04978 | Mineral absorption | 9 | 76 | 9.09 × 10^−5^ | 2.63 × 10^−3^ |
| ko05134 | Legionellosis | 13 | 143 | 4.61 × 10^−5^ | 2.63 × 10^−3^ |
| ko05204 | Chemical carcinogenesis | 11 | 110 | 7.45 × 10^−5^ | 2.63 × 10^−3^ |
| ko05222 | Small cell lung cancer | 14 | 175 | 9.64 × 10^−5^ | 2.63 × 10^−3^ |
| ko00480 | Glutathione metabolism | 9 | 98 | 6.30 × 10^−4^ | 1.20 × 10^−2^ |
| ko04115 | p53 signaling pathway | 12 | 142 | 7.00 × 10^−4^ | 1.20 × 10^−2^ |
| ko04217 | Necroptosis | 18 | 311 | 6.19 × 10^−4^ | 1.20 × 10^−2^ |
| ko04915 | Estrogen signaling pathway | 15 | 231 | 5.39 × 10^−4^ | 1.20 × 10^−2^ |
| ko04931 | Insulin resistance | 13 | 188 | 6.98 × 10^−4^ | 1.20 × 10^−2^ |
| ko05145 | Toxoplasmosis | 15 | 237 | 7.04 × 10^−4^ | 1.20 × 10^−2^ |
| ko04580 | Osteoclast differentiation | 14 | 215 | 7.98 × 10^−4^ | 1.28 × 10^−2^ |
| ko04974 | Protein digestion and absorption | 15 | 256 | 1.53 × 10^−3^ | 2.32 × 10^−2^ |
| ko01524 | Platinum drug resistance | 9 | 114 | 1.85 × 10^−3^ | 2.64 × 10^−2^ |
| ko04210 | Apoptosis | 18 | 372 | 1.93 × 10^−3^ | 2.64 × 10^−2^ |
| ko05132 | Salmonella infection | 12 | 193 | 2.72 × 10^−3^ | 3.54 × 10^−2^ |
| ko00982 | Drug metabolism − cytochrome P450 | 7 | 79 | 3.06 × 10^−3^ | 3.63 × 10^−2^ |
| ko04960 | Aldosterone−regulated sodium reabsorption | 7 | 79 | 3.06 × 10^−3^ | 3.63 × 10^−2^ |
| ko05133 | Pertussis | 12 | 201 | 3.79 × 10^−3^ | 4.31 × 10^−2^ |
| ko05225 | Hepatocellular carcinoma | 16 | 311 | 4.00 × 10^−3^ | 4.37 × 10^−2^ |
| ko05164 | Influenza A | 18 | 375 | 4.83 × 10^−3^ | 4.89 × 10^−2^ |
| ko05230 | Central carbon metabolism in cancer | 8 | 108 | 4.82 × 10^−3^ | 4.89 × 10^−2^ |
