# Supplementary figures and images for: Zebrafish Oxr1a Knockout Reveals Its Role in Regulating Antioxidant Defenses and Aging
Source: Genes (Basel). 2020 Sep 24;11(10):1118. doi: 10.3390/genes11101118 (PMC7598701; doi:10.3390/genes11101118)

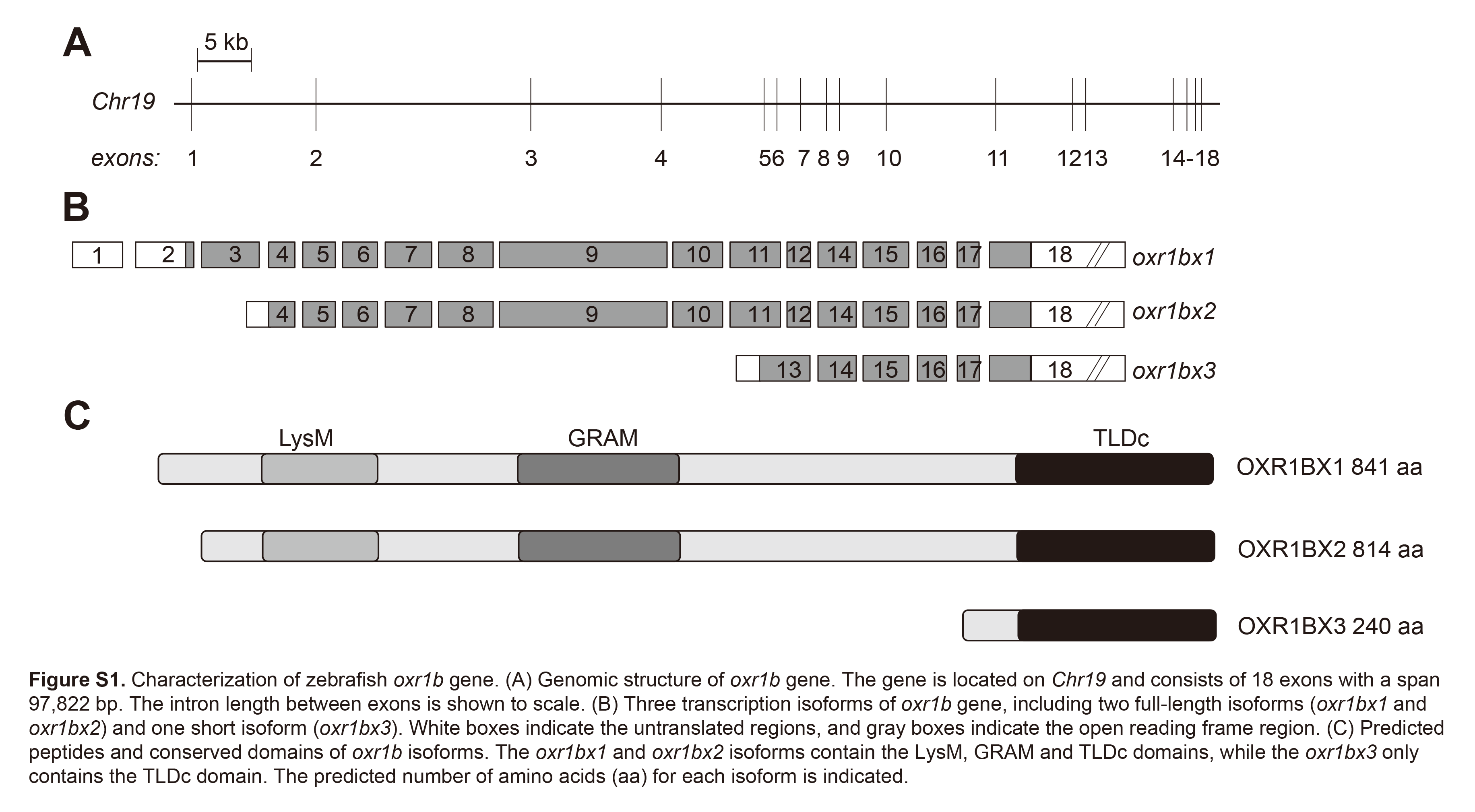

Supplement: Supplementary file 1 [file genes-11-01118-s001.zip › genes-921171-Supplementary files/Figure S1.tif]

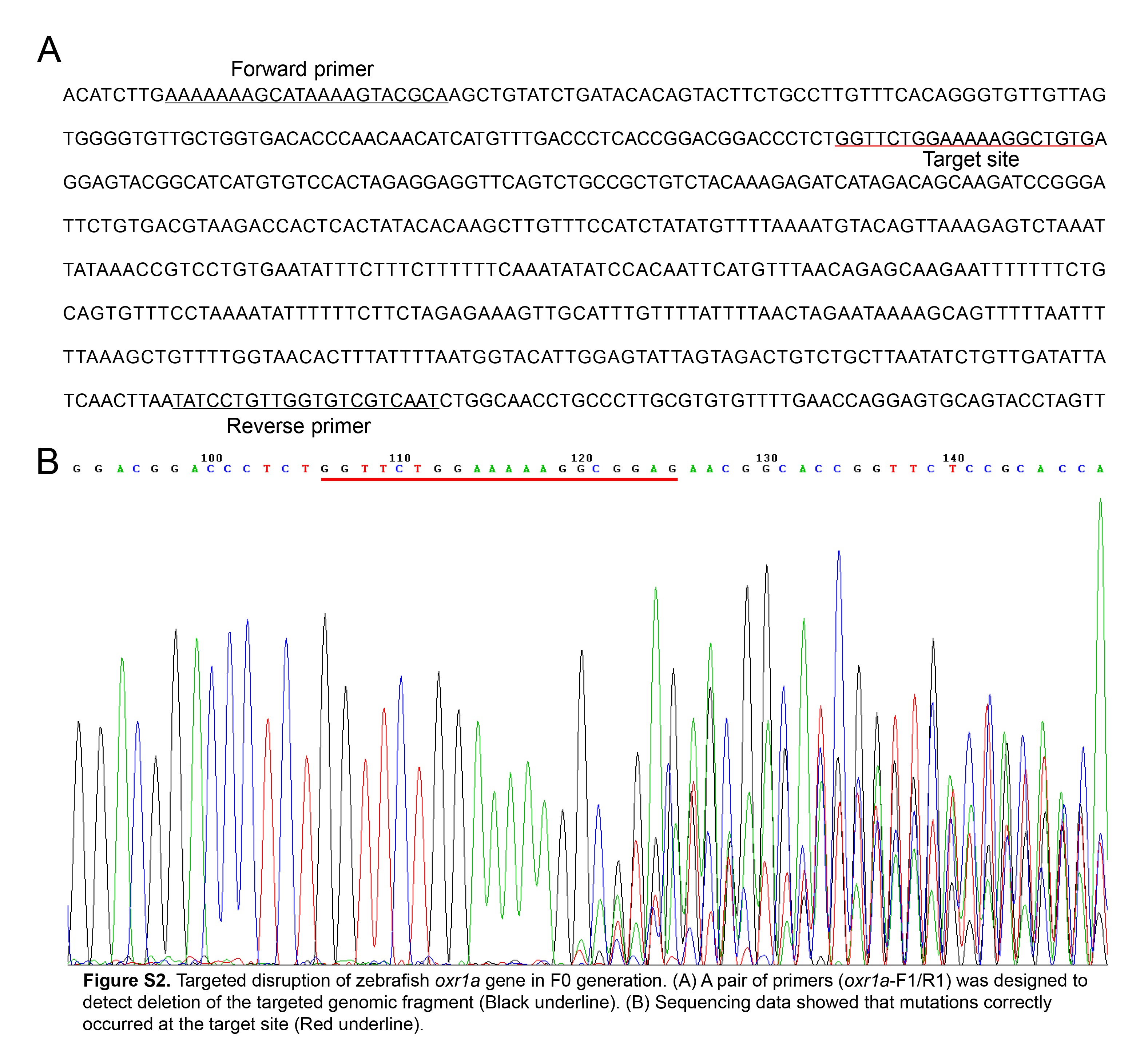

Supplement: Supplementary file 1 [file genes-11-01118-s001.zip › genes-921171-Supplementary files/Figure S2.tif]

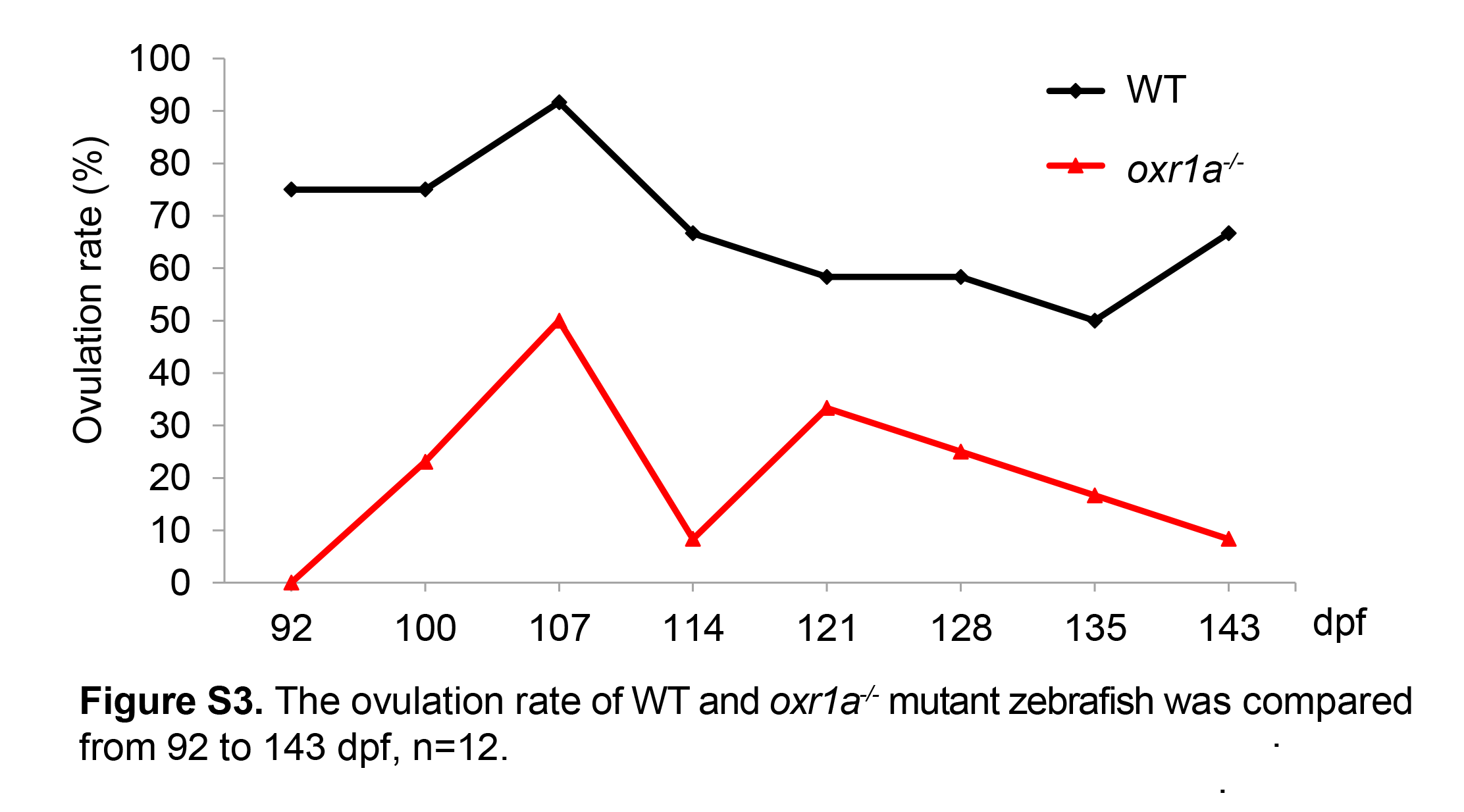

Supplement: Supplementary file 1 [file genes-11-01118-s001.zip › genes-921171-Supplementary files/Figure S3.tif]

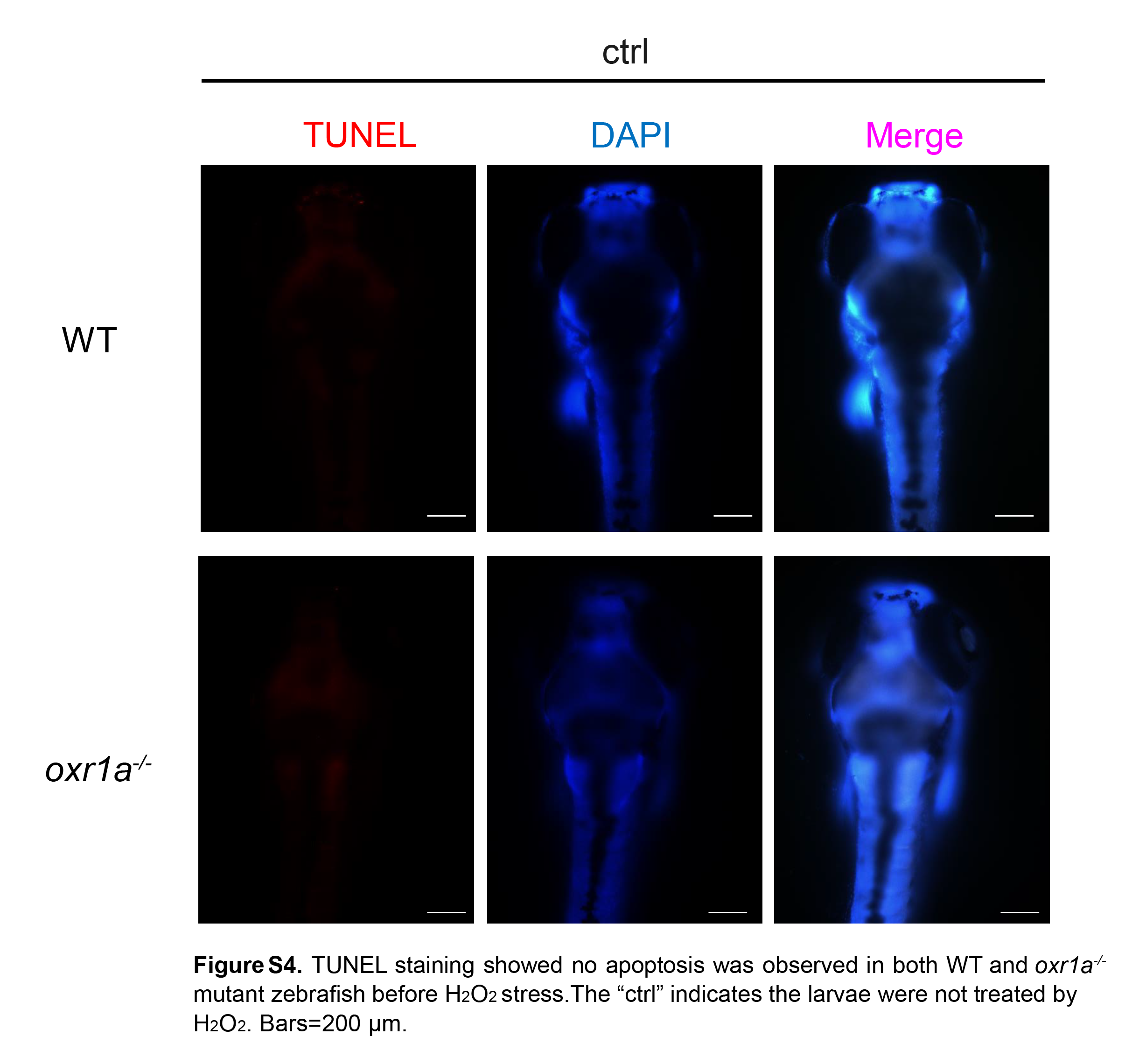

Supplement: Supplementary file 1 [file genes-11-01118-s001.zip › genes-921171-Supplementary files/Figure S4.tif]

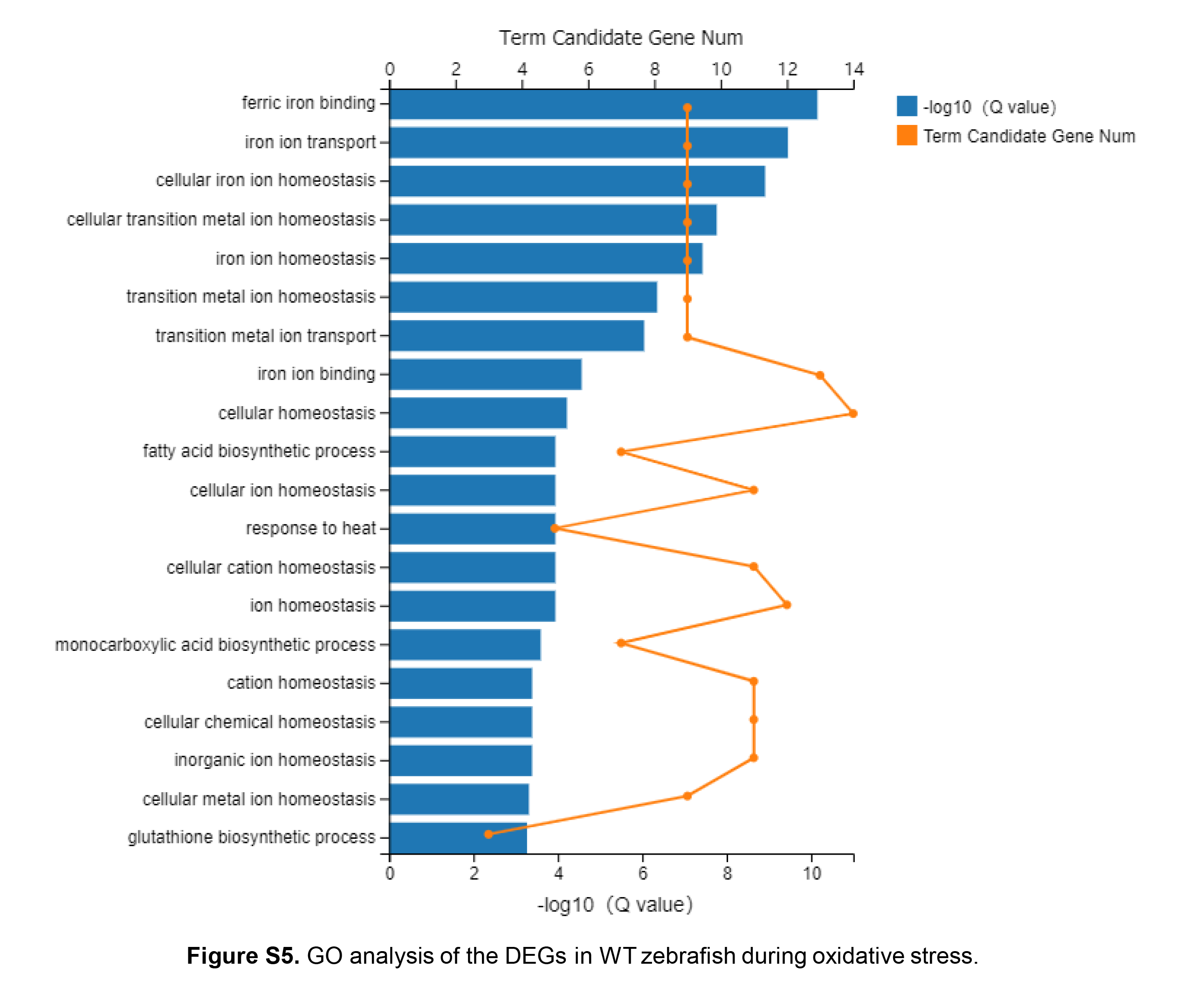

Supplement: Supplementary file 1 [file genes-11-01118-s001.zip › genes-921171-Supplementary files/Figure S5.tif]
